# Sense of mastery in first-episode psychosis—a one-year follow-up study

**DOI:** 10.3389/fpsyt.2023.1200669

**Published:** 2023-09-07

**Authors:** Maija Lindgren, Sebastian Therman, Tiina From, Jarmo Hietala, Heikki Laurikainen, Raimo K. R. Salokangas, Jaana Suvisaari

**Affiliations:** ^1^Mental Health, Public Health and Welfare, Finnish Institute for Health and Welfare, Helsinki, Finland; ^2^Department of Psychiatry, University of Turku and Turku University Hospital, Turku, Finland; ^3^Turku PET Centre, University of Turku, Turku, Finland

**Keywords:** mastery, psychosis, schizophrenia, remission, depression, social support

## Abstract

**Introduction:**

A sense of mastery refers to beliefs about having control over one’s life and has been found to protect health and buffer the effect of stressful experiences.

**Methods:**

We investigated sense of mastery in first-episode psychosis (FEP) patients and population controls at baseline and at one-year follow-up. Pearlin and Schooler’s Sense of Mastery scale was completed by 322 participants at baseline and by 184 participants at follow-up.

**Results:**

People having experienced FEP reported lower mastery than controls at both time points, but a modest increase was seen in patients at follow-up. The strongest correlates of high baseline mastery in FEP were lower depressive symptoms and higher perceived social support, whereas positive or negative psychotic symptoms did not associate with mastery. Current depressive symptoms also correlated with mastery at the follow-up point, and change in depressive symptoms correlated with change in mastery. Higher mastery at treatment entry predicted remission of psychotic symptoms one year later. Sense of mastery was also found to mediate the association of perceived social support with depressive symptoms.

**Discussion:**

The usefulness of mastery measures should be further tested for estimations of patient prognosis in early psychosis.

## 1. Introduction

A sense of mastery (1), indicating the experience of having control over events in one’s life, is a significant resource for wellbeing. Hence, mastery is often included as an important measure in public health surveys studying mental health (2–4). In the general population, a high sense of mastery has been found to improve both physical and mental health (5–7), even when controlling for various sociodemographic and social conditions (8). Furthermore, a stronger sense of mastery predicts self-rated health over the lifespan, also when objective physical health is taken into account (9, 10), and associates with lower mortality risk (11). Mastery may also mediate (12–14) or moderate (15) the relationship between vulnerability factors and mental health. Mastery beliefs are especially central when dealing with stressful events, and a sense of mastery over one’s surroundings can be a coping resource when encountering unpredictable circumstances and hazards in life (16). One such hardship can be a mental disorder.

Becoming severely mentally ill may generate deep feelings of powerlessness. When a severe mental illness emerges, limited control over one’s own condition can be exercised, and hospital treatment and even involuntary treatment may be required, understandably creating the perception that events relate to external rather than internal sources. Compared to the general population, individuals with psychotic disorder typically experience less control (17). However, positive resources of individuals living with psychotic disorders can be strengthened through empowerment, building up the experience of mastery in one’s own life and further increasing wellbeing. The recovery-oriented approach particularly emphasizes self-agency and control of individuals living with a psychotic disorder (18). Control beliefs are also related to hope in people with mental disorders (19).

Studies show that the perception that events in one’s life do not relate to one’s actions is widely associated with negative phenomena, both in the general population and among people with psychosis. In individuals with schizophrenia, feelings of happiness are linked more with a sense of mastery than symptom level (17), and mastery appears as a key predictor of quality of life (20, 21). A low sense of mastery has also been found to associate with more severe symptoms and more needs for care and support (22) as well as with lower occupational performance (23) in psychotic disorders. In one study of people with bipolar disorder, those with a higher sense of mastery showed fewer psychiatric symptoms but experienced a greater increase in depressive and anxiety symptoms during the COVID-19 pandemic (24). Using the related concept of *locus of control*, Harrow et al. (25) found that an internal locus of control (indicating high mastery) was associated with less depressed mood and increased recovery in schizophrenia.

Instead of being a stable construct, mastery is considered to change over the lifespan in response to life events (26). Little research has been conducted on the sense of mastery in first-episode psychosis (FEP), let alone on factors that contribute to changes in mastery in the course of early psychosis. In one longitudinal study, increasing mastery correlated with an increasing availability of social contact and easing of affective symptoms, but not positive or negative symptom-level changes (22). Social support is another source of coping with life stressors in addition to personal mastery (27), and whereas social support and mastery have been found to be linked in the population (3), this association in psychosis calls for further research.

Because of its wide correlations with everyday wellbeing and benefits to coping with life stressors, a sense of mastery is an important target for research in individuals with FEP. The aim of this study was to examine the correlates and the course of mastery in early psychosis. FEP as well as population control participants were investigated in two phases, 1 year apart. Higher mastery and positive changes in mastery during the follow-up period were hypothesized to be broadly associated with higher levels of social support and functioning, less severe symptomatology, and greater odds for remission.

## 2. Methods

### 2.1. Participants and study protocol

Participants were recruited from two early psychosis studies from geographically distinct Finnish sites, Helsinki (28) and Turku (29). Both studies recruited young adults with their first psychiatric treatment contact for affective or non-affective psychosis in hospitals and outpatient clinics in 2010–2017. In Helsinki, the inclusion criterion was defined as receiving a score ≥ 4 (moderate or higher) in unusual thought content (delusions) or hallucinations on the Brief Psychiatric Rating Scale, Expanded Version 4.0 (BPRS-E) (30). In Turku, the inclusion was based on psychotic disorder as defined by the Structured Interview for Prodromal Syndromes (SIPS) Presence of Psychotic Symptoms criteria (31), also using information from medical records. Exclusion criteria at both sites were substance-induced psychotic disorders or those caused by a general medical condition.

Age- and sex-matched control participants from the same catchment areas during the same period were recruited from the Finnish Population Information System, except a part of the Turku control sample, which was made up of students and personnel from Turku University of Applied Sciences. Psychotic disorders were an exclusion criterion, as were any chronic neurological or endocrinological diseases, or conditions preventing magnetic resonance imaging. Other mental health problems were allowed, and 18% of controls had a lifetime diagnosis of a mental disorder, most typically a depressive disorder. Control participants were assessed with the same measures and personnel as patients.

Patients were recruited as soon as possible after they had commenced treatment and were able to provide informed consent, as judged by the treating personnel. Hence, at the time of the first interview some FEP patients were already in remission from positive psychotic symptoms (in the last 7 days). The number of patients approached was not recorded, and refusal rates are thus unfortunately unavailable. Follow-up assessments were done after 12 months in Helsinki and after 9–12 months in Turku, with the follow-up time being 1 year on average (32). Invitations to the follow-up were sent via letters and text messages. Diagnoses were set by a senior psychiatrist based on the Structured Clinical Interview for the DSM-IV, Research Version (SCID-I/P) (33) as well as medical records.

All the participants gave written informed consent to participation. The data were analyzed using participant codes without personal identification information on the participants. The study protocols were approved by the Ethics Committees of the Hospital Districts of Helsinki and Uusimaa and Southwest Finland, respectively, and by the institutional review boards of the Finnish Institute for Health and Welfare and the University of Helsinki. The study was carried out in accordance with the Declaration of Helsinki (34).

### 2.2. Measures

#### 2.2.1. Questionnaire

To measure the sense of having control over the forces that affect one’s life, participants were asked to fill in the Sense of Mastery Scale (1) in both study phases. The scale has seven items and a four-point Likert response scale, from *Completely agree* to *Completely disagree*. Two of the items are in reverse. Example items include “What happens to me in the future mostly depends on me” and “Sometimes I feel that I am being pushed here and there in life” (reverse-scored) (see Supplementary material). The total sum score of the scale ranges between 7 and 28, with higher scores indicating greater levels of mastery. Psychometric studies on the Sense of Mastery have reported acceptable functioning of the scale (35, 36). In previous population studies, a high sense of mastery has been defined as a score of 23 or greater (3, 37).

In the same questionnaire, the Multidimensional Scale of Perceived Social Support (38) was used to assess perceived social support with 12 questions, using a frequency scale from *Never* (0) to *Always* (4). Acceptable psychometric properties of the social support questionnaire have been reported in clinical samples (39–41). The 1978 version of the Beck Depression Inventory (BDI) (42) was used to measure current depressive symptoms with 21 statements on a four-point severity-rating scale. BDI shows high reliability and validity in individuals with schizophrenia (43, 44). The Beck Anxiety Inventory (BAI) (45) assessed primarily somatic anxiety symptoms over the past 7 days with 21 items using a four-point severity-rating scale. BAI has been shown to have adequate psychometric properties in people with psychosis (46).

#### 2.2.2. Interview

Interviews were conducted by trained research staff with the 24-item version of BPRS-E that uses scales of 1–7, with higher score indicating more severe symptoms (30), or the Positive and Negative Syndrome Scale (PANSS) (47).

Similarly to previous works combining participants from the two sites (32, 48), symptom equivalents for the FEP subgroup interviewed with PANSS instead of BPRS were used: PANSS item P3 Hallucinatory Behavior was considered to correspond to BPRS item 10 Hallucinations, P1 Delusions in PANSS to item 11 Unusual Thought Content in BPRS, and PANSS P2 Conceptual Disorganization to BPRS item 15 Conceptual Disorganization. Item scores of hallucinations, unusual thought content, and conceptual disorganization were summed to form a positive psychotic symptom score (sum score range 3–21).

Furthermore, PANSS N1 Blunted Affect was considered to correspond to BPRS item 16 Blunted Affect, used to assess negative symptoms (range 1–7).

Symptomatic remission was defined using the criteria of Andreasen et al. (49) at the time of the follow-up interview.

Level of functioning was assessed with the Social and Occupational Functioning Assessment Scale (SOFAS) (50) on a scale of 1–100 in each study phase.

### 2.3. Statistical analyses

IBM SPSS Statistics for Windows, version 28 was used for statistical analyses, with the limit for statistical significance set at *p* < 0.05, except in the correlation analyses it was set at *p* < 0.01 to compensate for multiple testing. The participant groups were compared with the Mann–Whitney *U* test. Group differences, as expressed as Vargha & Delaney *Â* effect sizes (stochastic superiority) (51), were calculated from Mann–Whitney *U* values: *U* / (*n*1 × *n*2). The *Â* value is the probability that a random group A member has a value above that of a random group B member, with split ties: *P*(A > B) + ½ × *P*(A = B). *Â* values thus range from 0 to 1, where 0.5 means no group difference and 1 would indicate that all group A members have values above those of the group B members.

Spearman rank-order correlations (*r*) were used to examine the associations between mastery and other continuous variables of interest at both time-points. Change in mastery over the year was calculated (Mastery follow-up total score—Mastery baseline total score) and was also correlated with change in other scales.

Linear regression models were conducted predicting mastery in FEP. We report unstandardized *B* coefficients with 95% confidence intervals (CI) as well as *R*^2^ and adjusted *R*^2^ values, and standardized *β* values to allow for comparability between models. Secondly, a logistic regression model predicting remission with baseline mastery level was performed, reporting odds ratios (*OR*) with 95% CI. All predictors were entered in the models simultaneously.

*Post-hoc* mediation models were performed to see whether mastery mediated the relationship between social support and depressive symptoms in FEP. This was performed with the SPSS macro PROCESS (52), using model 4 with 5,000 bootstrap samples. The predictor was baseline social support, the dependent variable baseline depressive symptoms, the mediator baseline sense of mastery, and the covariates were age, sex, education, and baseline anxiety, positive, and negative symptoms, and baseline functioning level.

## 3. Results

### 3.1. Participants and their mastery levels

A total of 333 participants with mastery data were included, 186 in the FEP and 147 in the control group. Of them, 179 FEP participants and 143 controls filled in the Sense of Mastery Scale at baseline. Table 1 shows the demographic information of the participants. A year later, mastery data was available from 89 FEP and 95 control participants. FEP participants with one-year follow-up mastery data had higher baseline education and SOFAS scores compared to those without one-year follow-up data, while there were no baseline mastery, age, social support, or symptom differences between the groups attending or not attending follow-up. In population controls there were no differences in these variables between those attending or not attending follow-up.

**Table 1:** Participants in the groups and their mastery levels, along with other scales.

	First-episode psychosis, *n* = 186		Population control, *n* = 147		Group difference effect size^d^ *Â*
	*M* (SD) or *n* (%)	Range	*M* (SD) or *n* (%)	Range	
Females	76 (40.9%)		72 (49.0%)		
Affective psychotic disorder^a^	39 (21.0%)		–	–	–
**Baseline**					
Age	26.4 (5.8)	18–46	27.4 (6.0)	19–49	0.45
Education (years, self-reported)	13.8 (3.0)	8–24	15.6 (2.4)	11–24	0.29
Mastery (*n*_FEP_ = 179, *n_C_* = 143)	19.3 (4.7)	7–28	24.2 (3.1)	14–28	0.19
Social support	36.2 (12.7)	0–60	45.5 (9.3)	12–73	0.28
BAI	15.4 (12.4)	0–60	2.8 (3.9)	0–25	0.87
BDI	13.4 (11.1)	0–54	2.9 (4.6)	0–31	0.85
Positive symptoms^b^	7.8 (3.3)	3–15	3.1 (0.3)	3–5	0.92
Negative symptoms^c^	2.2 (1.1)	1–5	1.0 (0.2)	1–3	0.79
SOFAS	47.1 (13.3)	20–90	89.1 (5.4)	67–100	0.01
**Follow-up**					
Mastery (*n*_FEP_ = 89, *n_C_* = 95)	20.8 (4.8)	9–28	24.2 (3.7)	7–28	0.29
Mastery change (*n*_FEP_ = 82, *n_C_* = 91)	2.1 (4.1)	−8 to 14	−0.1 (3.3)	−15 to 7	0.64
Social support	39.6 (14.3)	4–60	46.0 (8.4)	20–60	0.37
BAI	7.8 (8.4)	0–30	2.9 (4.4)	0–25	0.67
BDI	9.3 (9.8)	0–42	3.0 (5.0)	0–26	0.70
Positive symptoms^b^	4.7 (2.5)	3–14	3.0 (0.2)	3–4	0.73
Negative symptoms^c^	2.1 (1.2)	1–6	1.0 (0.2)	1–2	0.78
SOFAS	58.8 (16.7)	20–90	87.6 (7.4)	45–95	0.05

BAI, Beck Anxiety Inventory.

BDI, Beck Depression Inventory.

SOFAS, Social and Occupational Functioning Assessment Scale.

a Psychotic depression or bipolar disorder; the rest were diagnosed with nonaffective psychotic disorder.

b Sum of BPRS (or PANSS) Hallucinations, Delusions, and Conceptual Disorganization.

c BPRS (or PANSS) Blunted Affect.

d Vargha & Delaney Â: *p*(*X* > *Y*) + 0.5 × *p*(*X* = *Y*).

**Figure 1:**
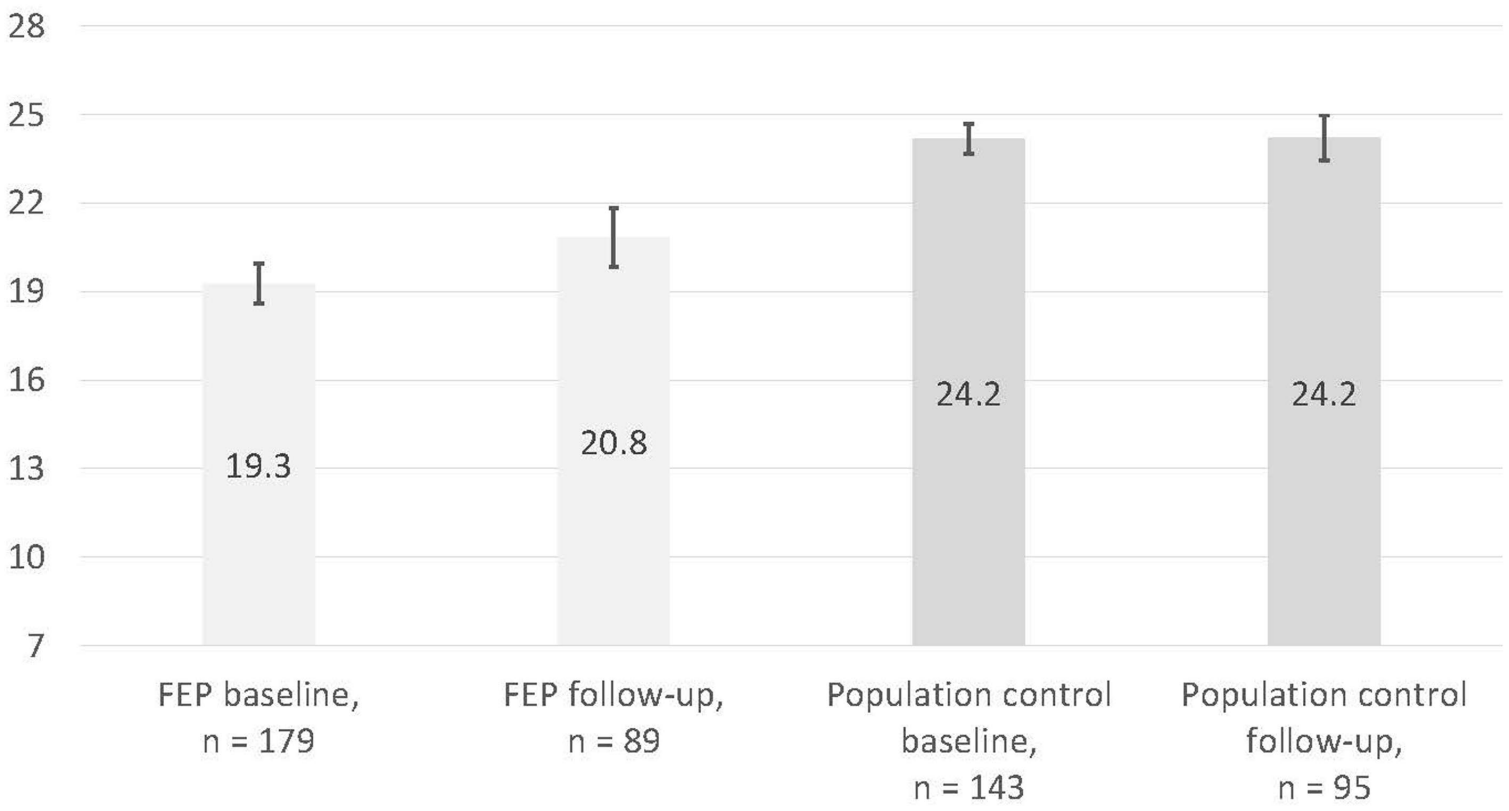
Sense of mastery means and 95% confidence intervals in the study groups. Sense of mastery mean change over the follow-up was 2.1 ± 4.1 in 82 FEP participants and −0.1 ± 3.3 in 91 controls.

Table 1 and Figure 1 show the mastery levels in the study groups. Sense of mastery was higher in population controls at both time points compared to the FEP group (*p* < 0.001). There were no age or sex differences in the mastery sum scale. Education years did not correlate with mastery when the FEP and control groups were studied separately. In the FEP group, there were no significant differences in mastery between those diagnosed with affective psychosis (psychotic depression or bipolar disorder; *n* = 38, baseline mastery *M* 19.5, SD 4.4) and those with nonaffective psychotic disorder (other psychotic disorders; *n* = 141, *M* 19.2, SD 4.7). Persons with FEP also reported lower social support and stronger depressive symptoms than the control group.

During the follow-up, average mastery levels increased somewhat in FEP participants and remained at the same level in controls, but individual differences were large (Table 1, Supplementary Figure S1). Among controls, older participants had more positive changes in mastery during the year (*r* = 0.21, *p* = 0.043), while the change in mastery was not otherwise linked to sociodemographic factors.

### 3.2. Correlates of baseline mastery

Looking at cross-sectional associations at baseline, mastery in persons with FEP correlated widely with other scales, most strongly with less depressive and anxiety symptoms and stronger perceived social support, and only weakly with less psychotic symptoms (Table 2). It is of note that baseline mastery also correlated negatively with follow-up scales such as depression, anxiety, and positive and negative symptoms.

**Table 2:** Correlations between mastery scale and other scales in FEP participants.

	Mastery baseline		Mastery follow-up		Mastery change	
	*r*	*p*	*r*	*p*	*r*	*p*
Positive symptoms, B	−0.18	0.022	−0.01	0.961	0.11	0.350
Positive symptoms, F	**−0.33**	<0.001	**−0.42**	<0.001	−0.03	0.762
Positive symptoms, Change	0.08	0.429	−0.14	0.19	−0.06	0.606
Negative symptoms, B	−0.19	0.013	−0.10	0.357	−0.03	0.823
Negative symptoms, F	**−0.31**	0.001	**−0.36**	0.001	0.04	0.757
Negative symptoms, Change	−0.17	0.065	−0.27	0.012	0.04	0.698
Social support, B	**0.43**	<0.001	**0.49**	<0.001	−0.06	0.587
Social support, F	**0.36**	0.001	**0.46**	<0.001	0.10	0.390
Social support, Change	−0.21	0.057	0.03	0.799	0.21	0.053
BAI, B	**−0.44**	<0.001	−0.17	0.128	0.13	0.239
BAI, F	**−0.41**	<0.001	**−0.46**	<0.001	−0.06	0.587
BAI, Change	−0.05	0.637	−0.20	0.074	−0.18	0.105
BDI, B	**−0.65**	<0.001	**−0.44**	<0.001	0.20	0.077
BDI, F	**−0.55**	<0.001	**−0.72**	<0.001	−0.20	0.075
BDI, Change	0.19	0.091	−0.18	0.108	**−0.40**	<0.001
SOFAS, B	0.00	0.971	0.04	0.736	0.09	0.405
SOFAS, F	**0.25**	0.007	**0.34**	0.001	0.02	0.850
SOFAS, Change	0.21	0.024	**0.29**	0.007	−0.04	0.731

B, Baseline.

F, Follow-up.

r, Rank-order correlation.

p, Significance level.

BAI, Beck Anxiety Inventory.

BDI, Beck Depression Inventory.

SOFAS, Social and Occupational Functioning Assessment Scale.

Significant associations (*p* < 0.01) are in boldface.

In the controls, less depressive and anxiety symptoms and higher perceived social support and functioning correlated with stronger mastery (Supplementary Table S1).

The baseline mastery score was higher among patients with follow-up remission (*n* = 60; mastery mean 20.4 ± 4.7) compared to those who had not remitted in the follow-up assessment (*n* = 58; 17.9 ± 4.6; Mann–Whitney *U* = 2,280, *p* = 0.004, *Â* = 0.66). In a logistic regression model, baseline mastery level predicted remission (OR = 1.2, 95% CI 1.0–1.3, *p* = 0.013), when controlling for baseline variables of age, sex, education, diagnosis group, positive and negative symptoms, and functional level.

In a linear regression model using the baseline measures of the FEP group, lower depressive symptoms (*B* = −0.23, 95% CI −0.29 to −0.16, *β* = −0.56, *p* < 0.001), higher perceived social support (*B* = 0.07, 95% CI 0.02–0.12, *β* = 0.19, *p* = 0.006), and fewer education years (*B* = −0.25, 95% CI −0.46 to −0.04, *β* = −0.17, *p* = 0.021) predicted mastery, when the model also included age, sex, diagnosis group, anxiety, positive, and negative symptoms, as well as functional level (model *R*^2^ 0.48, adjusted *R*^2^ 0.45).

### 3.3. Correlates of follow-up mastery

It can be seen in Table 2 that baseline as well as follow-up scales correlated with follow-up mastery. In people having experienced a psychotic episode, low perceived social support and more severe depressive symptoms at both timepoints were linked to low personal mastery at follow-up. However, only follow-up (current) anxiety correlated with follow-up mastery, while baseline anxiety, positive symptoms, and SOFAS did not. Remission was also connected with higher mastery (*U* = 1,301, *p* = 0.004, *Â* = 0.68). In controls, current anxiety and depressive symptoms correlated with mastery at the follow-up (Supplementary Table S1).

In a regression model predicting follow-up mastery with follow-up scales in the FEP group, fewer depressive symptoms (*B* = −0.29, 95% CI −0.41 to −0.17, *β* = −0.59, *p* < 0.001) and lower number of education years (*B* = −0.32, 95% CI −0.61 to −0.02, *β* = −0.21, *p* = 0.037) were significant predictors, when controlling for age, sex, diagnosis group; anxiety, positive, and negative symptoms; social support, and functional level (model *R*^2^ 0.56, adjusted *R*^2^ 0.50).

### 3.4. Correlates of change in mastery during follow-up

**Figure 2:**
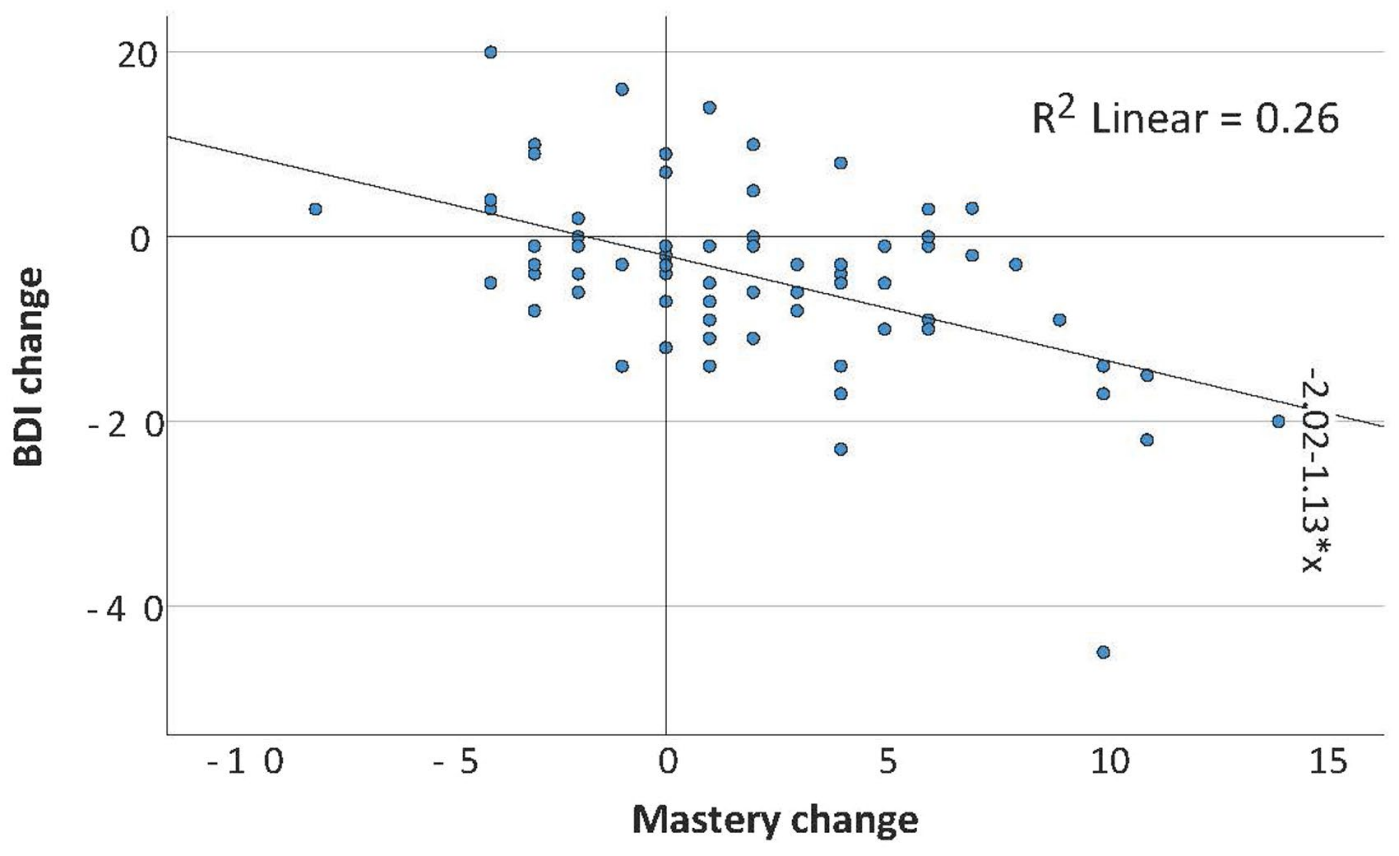
FEP group change in mastery vs. change in depressive symptoms scores over the follow-up year, with regression line.

Correlations between change in mastery and change in other scales over the follow-up year are presented in Table 2 (FEP) and Supplementary Table S1 (controls). In FEP, only change in depressive symptoms correlated negatively with change in mastery. Figure 2 shows the associations between these two.

### 3.5. Mastery as a mediator

As sense of mastery was most strongly associated with depressive symptoms and social support, we next investigated the relationship between these three variables at baseline with *post-hoc* mediation analyses in 158 people with psychosis with all needed variables available.

**Figure 3:**
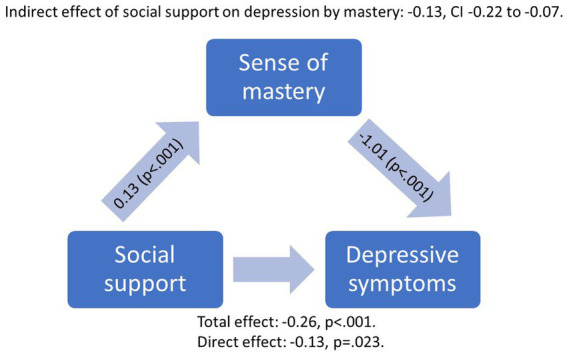
Mediation model with unstandardized coefficients of direct and indirect effects of perceived social support on depression by mastery at baseline, when controlling for age, sex, education years, functioning level, and anxiety, positive, and negative symptom levels.

Figure 3 shows the coefficients of direct and indirect effects of the model. Social support was linked with lower levels of depressive symptoms both directly and indirectly through a higher level of sense of mastery (Effect −0.13; 95% CI −0.22 to −0.07), controlling for age, sex, education, anxiety, positive, and negative symptoms, and functioning level. Of the relationship between social support and depressive symptoms, 51.6% was mediated by mastery level.

## 4. Discussion

There has been a shift from studying merely the symptomatic presentation of psychotic disorder toward monitoring and promoting the psychosocial resources and mental wellbeing of individuals living with psychotic disorder. Sense of mastery reflects the extent to which respondents regard their life circumstances as being under their personal control. These control beliefs over one’s life were studied in individuals with their first episode of psychosis as well as matched control participants. Sense of mastery is conceptually close to agency (53), self-efficacy (54), and sense of coherence (55). However, sense of mastery is a separate construct, most commonly measured with the seven-item Sense of Mastery scale by Pearlin and Schooler (1).

Individuals with FEP reported lower mastery (mean score 19.3) compared to the population controls (mean score 24.2). The level of mastery in FEP was in line with an earlier study with people with schizophrenia living in the Swedish community (mean 19) (22), but higher than in outpatients with schizophrenia in Taiwan (mean 17.2) (21). Using the previously suggested ≥23 score cutoff, 27% of the FEP participants and 72% of the controls reported “high mastery.”

At the one-year follow-up, FEP participants still reported a lowered sense of mastery but there was a two-point average increase in the mastery score at follow-up (mastery mean 20.8). Therefore, 42% of the FEP participants and 72% of the controls reported “high mastery” at follow-up. In controls, mastery on average remained stable, in line with earlier results with a longer follow-up in young adults (9).

Educational level, indicating socioeconomical status, has been positively associated with mastery in previous general population studies (3, 56). In one study, mastery mediated the link between education and psychological distress, with the authors suggesting that education might promote empowerment (13). In the current study, age, sex, or education were not correlated with mastery but it should be noted that our young adult participants were often students who had not yet finished their studies. This is also why we used self-reported years spent in education rather than achieved educational level. In the regression models, *fewer* education years were a significant predictor of higher mastery in FEP, along with milder depressive symptoms. We have previously reported that in the Helsinki FEP sample, higher cognitive performance was linked with affective symptoms at the time of the first psychotic episode (57). This may indicate that affective symptoms are adequate reactions to getting severely ill and markers of an intact cognitive performance (58). This may be associated with insight, which is also positively linked with cognitive capacity (59, 60). During the first psychotic episode, losing the sense of control may possibly be especially pronounced among people with high education, and is an important theme that should be addressed in the psychosocial treatment of these patients (61). Building a new identity is part of the recovery (62, 63), and individual differences such as level of mastery should be acknowledged in planning rehabilitation.

Mastery was negatively associated with psychopathology, especially depressive symptoms, which was in line with our hypothesis and repeated previous findings both in individuals with major psychiatric disorders (22) and in the general population (5, 12). However, positive and negative symptoms were not strongly related with mastery in FEP, though it should be noted that the variation in positive symptoms was rather narrow due to inclusion criteria. Remission of positive and negative psychotic symptoms at follow-up was predicted by higher mastery at baseline, even when controlling for baseline symptom and functional levels, in line with Harrow et al.’s (25) finding of internal locus of control predicting recovery in schizophrenia. While mastery has been found to predict remission of depression (64) and outcome in anxiety and depressive disorders (65), the usefulness of mastery measures for estimations of patient prognosis specifically in early psychosis calls for further research.

An increasing sense of mastery over the year following the first episode was associated with alleviation of depressive symptoms but not with changes in other symptoms, functional level, or social support. Also, in some previous studies changes in mastery were negatively correlated to changes in affective symptoms in schizophrenia (22) and in the general population (27). In one study, there were associations between mastery and depression cross-sectionally, longitudinally, and even intergenerationally between mothers and offspring (66).

In addition to symptoms, perceived social support was significantly related to mastery. In earlier general population studies, social support was the strongest predictor of mastery (3), and changes in mastery were predicted by baseline social support (27). The association between mastery and social support may be reciprocal, with perceived social support enhancing the sense of mastery, but mastery also increasing social engagement (67), using social resources effectively, or perception of stronger support when coping with stressful situations. Both mastery and social support are considered important resources in coping and resilience, which protect the individual when facing stress. A previous study found that in mothers with a serious mental illness who had a high sense of mastery, social support was more beneficial compared to mothers with a low sense of mastery (68). The role of strong social support may be especially central in facing mental health problems as it predicts better outcomes (69). Both social support and mastery mediated the association between childhood trauma and risk of major depression in a cohort study (70). In individuals with schizophrenia, higher levels of both mastery and social support were linked to higher levels of quality of life (21).

In our *post-hoc* analyses, we found that having a low level of baseline social support transmitted its effect on depression through mastery, as over half of the association between social support and depressive symptoms was mediated via sense of mastery. Similar results have been reported using general population repondents: in university students, the positive relationship between social support and mental health was mediated by mastery (16), and in a cohort study, mastery mediated the link between stressors and depressive symptoms (12). However, the direction of causality is still unclear and merits further study in larger longitudinal samples.

### 4.1. Strengths and limitations

Mastery levels of the FEP participants were first assessed soon after the initiation of treatment, offering information on the clinical state of often still having symptoms at a psychotic level. A longitudinal design was used, enabling us to study the possible change in mastery over 1 year, although there was drop-out due to not reaching the person, or inability or unwillingness to participate. Two individual samples offered a larger dataset, but combining two independent cohorts resulted in some differences in the measures and protocols. In a naturalistic design, the duration from treatment entry to taking part in the study may vary, as is also the case concerning the duration of untreated psychosis. A matched control sample was available, providing perspective on mastery and its correlates in the general population compared with the context of serious mental illness.

Several further limitations also deserve a mention. Of the various factors possibly linked with mastery, we did not analyze insight or stigma (21). We also did not include trauma experiences, which may contribute to sense of mastery. On the other hand, mastery may affect coping with stress (16). We have previously reported on adverse childhood experiences in the Helsinki subcohort, where only having been bullied was associated with a lower sense of mastery, and only in male FEP patients; and the severity of overall adversity was correlated with lowered sense of mastery in male controls (71). In future studies, occupational outcomes in relation to mastery could also be investigated further.

## 5. Conclusion

This study investigated positive resources in individuals having experienced psychosis. According to many studies, there are health benefits associated with a greater sense of mastery both in the general population (9–11, 72) and in people with psychosis. However, few studies have been conducted on mastery in early psychosis. As people having experienced psychosis are a vulnerable group, achieving a sense of mastery may be particularly crucial (21) as a distress buffer. How individuals with psychosis think about themselves, their life, and their illness may relate to symptom severity, need for care, and quality of life (20, 22). Following FEP, identity change and reconstruction are a part of recovery (62). Autonomy may be inhibited during the course of severe mental illness and strengthening the sense of personal control through psychosocial interventions may promote mental wellbeing.

Assessing personal positive resources and strengthening control beliefs should thus be emphasized in the treatment of psychotic disorders, instead of merely paying attention to the reduction of psychotic symptoms. In the current study, perceived powerlessness was related especially to depressive symptoms and lack of social support, instead of psychosis symptoms or more objective measures at a functional level. These results can be seen to be in line with the personal recovery and recovery-oriented approaches (18, 73). The finding that depression is attached to experiences of mastery further emphasizes the relevance of mastery. Depressive symptoms predict subsequent suicidal thoughts and behaviors (74) and worse functional outcome in FEP (75), highlighting the significance of depressive symptoms worsening quality of life among people living with psychosis. In our study, social support seemed to strengthen mastery and, through that, ease depressive symptoms. As sense of mastery also predicted later clinical remission, mastery may be an important target in the mental health promotion of individuals with a first psychotic episode. It is important to pay attention to strengthening the sense of control as a goal of psychiatric rehabilitation.

## Data availability statement

The datasets presented in this article are not readily available because data are from the Helsinki Early Psychosis Study at the Finnish Institute for Health and Welfare and from the Turku Early Psychosis Study at the Hospital District of Southwest Finland. Sharing of the data is possible in research collaborations if it is in agreement with the consent given by the participants and with the General Data Protection Regulation (GDPR) and other applicable law. Collaborations require a separate agreement and local ethical committee approval. Requests to access the datasets should be directed to maija.lindgren@thl.fi.

## Ethics statement

The studies involving humans were approved by The Ethics Committees of the Hospital Districts of Helsinki and Uusimaa and Southwest Finland, the institutional review boards of the Finnish Institute for Health and Welfare and the University of Helsinki. The studies were conducted in accordance with the local legislation and institutional requirements. The participants provided their written informed consent to participate in this study.

## Author contributions

JH, RS, and JS: study planning and leading. TF: data management and coordination in Turku. ML, ST, JH, HL, and JS: analysis plan. ML: statistical analyses, literature searches, and first draft of the manuscript. All authors contributed to the article and approved the submitted version.

## Funding

This work was supported by the Academy of Finland (grants #278171 and #323035 to JS and #26080794 to JH), the Sigrid Jusélius Foundation (JS), Finnish Cultural Foundation (JS), Turku University Hospital (grants #30019 to HL and P3848 to JH), and Social Insurance Institution of Finland (#83/26/2021 to JH). The funding sources had no involvement in study design; in the collection, analysis and interpretation of data; in the writing of the paper; and in the decision to submit it for publication.

## Conflict of interest

The authors declare that the research was conducted in the absence of any commercial or financial relationships that could be construed as a potential conflict of interest.

## Publisher’s note

All claims expressed in this article are solely those of the authors and do not necessarily represent those of their affiliated organizations, or those of the publisher, the editors and the reviewers. Any product that may be evaluated in this article, or claim that may be made by its manufacturer, is not guaranteed or endorsed by the publisher.
