# Supplementary material for: Sense of mastery in first-episode psychosis—a one-year follow-up study
Source: Front Psychiatry. 2023 Sep 7;14:1200669. doi: 10.3389/fpsyt.2023.1200669 (PMC10512717; doi:10.3389/fpsyt.2023.1200669)
Supplement: Supplementary file 1 [file Data_Sheet_1.PDF]

Supplementary material /
Sense of mastery in first-episode psychosis—A one-year follow-up study

Items in the Sense of Mastery scale (Pearlin and Schooler, 1978)

a) I have little control over things that happen to me.
b) There's no way I can solve some of the problems I have.
c) There's little I can do to change most of the important things in my life.
d) Most of the time, I feel helpless when dealing with problems of life.
e) Sometimes I feel that I am being pushed here and there in life.
f) What happens to me in the future mostly depends on me.
g) I can do anything when I put my mind to it.

Response scale: Completely agree / Somewhat agree / Somewhat disagree / Completely disagree.

Supplementary Table. Correlations between mastery scale and other scales in controls

	Mastery Baseline		Mastery Follow-up		Mastery Change	
	r	*p*	*r*	*p*	*r*	*p*
Social support, B	**.26**	.002	.27	.011	.07	.497
Social support, F	.00	.975	.18	.081	.15	.163
Social support, Change	-.16	.140	-.04	.687	.09	.405
BAI, B	**-.49**	<.001	**-.44**	<.001	.00	.969
BAI, F	**-.38**	<.001	**-.34**	<.001	-.03	.806
BAI, Change	.17	.123	.12	.252	-.04	.725
BDI, B	**-.48**	<.001	**-.37**	<.001	-.05	.665
BDI, F	**-.32**	.002	**-.32**	.002	-.01	.916
BDI, Change	.07	.544	-.07	.499	-.07	.500
SOFAS, B	**.23**	.007	**.26**	.010	.04	.691
SOFAS, F	.22	.028	**.32**	.001	.10	.365
SOFAS, Change	-.06	.532	.13	.206	.15	.160

B, baseline
F, follow-up
r, rank-order correlation
p, significance level
BAI, Beck Anxiety Inventory
BDI, Beck Depression Inventory
SOFAS, Social and Occupational Functioning Assessment Scale
Significant associations ($p < 0.01$) are in boldface.

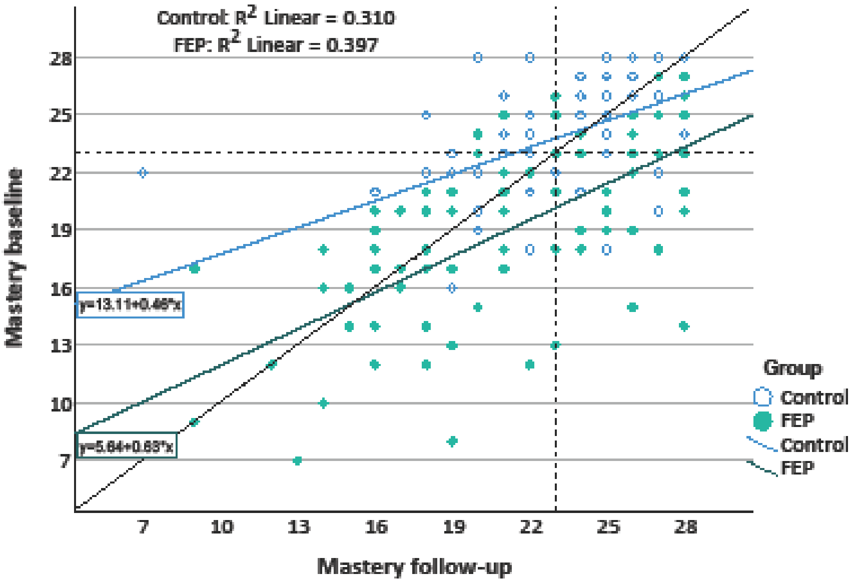

Supplementary Figure. Mastery levels at baseline and follow-up in the two study groups. The dotted line represents the "high mastery" cutoff of a score of ≥ 23.
